# Effects of neuromuscular electrical stimulation and voluntary commands on the spinal reflex excitability of remote limb muscles

**DOI:** 10.1007/s00221-019-05660-6

**Published:** 2019-10-10

**Authors:** Tatsuya Kato, Atsushi Sasaki, Hikaru Yokoyama, Matija Milosevic, Kimitaka Nakazawa

**Affiliations:** 1 Department of Life Sciences, Graduate School of Arts and Sciences, The University of Tokyo, 3-8-1 Komaba, Meguro-ku, Tokyo, 153-8902 Japan; 2 Japan Society for the Promotion of Science, 5-3-1 Kojimachi, Chiyoda-ku, Tokyo, 102-0083 Japan; 3 Department of Electrical and Electronic Engineering, Tokyo University of Agriculture and Technology, 2-24-16 Nakacho, Koganei-shi, Tokyo, 184-8588 Japan; 4 Rehabilitation Engineering Laboratory, Lyndhurst Centre, Toronto Rehabilitation Institute, University Health Network, 520 Sutherland Drive, Toronto, ON M4G 3V9 Canada; 5 Graduate School of Engineering Science, Department of Mechanical Science and Bioengineering, Osaka University, 1-3 Machikaneyama, Toyonaka, 560-8531 Japan

**Keywords:** Inter-limb connectivity, Spinal reflex, Neuromuscular electrical stimulation (NMES), Voluntary contraction

## Abstract

It is well known that contracting the upper limbs can affect spinal reflexes of the lower limb muscle, via intraneuronal networks within the central nervous system. However, it remains unknown whether neuromuscular electrical stimulation (NMES), which can generate muscle contractions without central commands from the cortex, can also play a role in such inter-limb facilitation. Therefore, the objective of this study was to compare the effects of unilateral upper limb contractions using NMES and voluntary unilateral upper limb contractions on the inter-limb spinal reflex facilitation in the lower limb muscles. Spinal reflex excitability was assessed using transcutaneous spinal cord stimulation (tSCS) to elicit responses bilaterally in multiple lower limb muscles, including ankle and thigh muscles. Five interventions were applied on the right wrist flexors for 70 s: (1) sensory-level NMES; (2) motor-level NMES; (3) voluntary contraction; (4) voluntary contraction and sensory-level NMES; (5) voluntary contraction and motor-level NMES. Results showed that spinal reflex excitability of ankle muscles was facilitated bilaterally during voluntary contraction of the upper limb unilaterally and that voluntary contraction with motor-level NMES had similar effects as just contracting voluntarily. Meanwhile, motor-level NMES facilitated contralateral thigh muscles, and sensory-level NMES had no effect. Overall, our results suggest that inter-limb facilitation effect of spinal reflex excitability in lower limb muscles depends, to a larger extent, on the presence of the central commands from the cortex during voluntary contractions. However, peripheral input generated by muscle contractions using NMES might have effects on the spinal reflex excitability of inter-limb muscles via spinal intraneuronal networks.

## Introduction

More than a century has passed since Ernst Jendrássik found that clenching the teeth or the fists facilitates tendon taps (i.e., tendon jerk reflex) in the lower limbs (Jendrássik 1883). Nowadays, facilitation of tendon jerk reflexes by the contraction of remote muscles is known as Jendrássik maneuver, and is widely used in clinical diagnosis of neurologically impaired patients (Gregory et al. 2001). Jendrássik maneuver is observed not only in the tendon jerk reflex modulation, but also in the electrically induced Hoffman reflex (H-reflex) (Landau and Clare 1964; Bussel et al. 1978). Although the mechanism of facilitation was initially thought to be related to the fusimotor system (Burg et al. 1973; Ribot et al. 1986), reduction of presynaptic inhibition onto Ia afferent terminals recently emerged as the leading candidate to explain the Jendrássik maneuver mechanism (Dowman and Wolpaw 1988; Zehr and Stein 1999; Gregory et al. 2001; Tazoe et al. 2005). Inter-limb (i.e., between upper and lower limb) reflex evoked by electrical stimuli is thought to be modulated via the long propriospinal reflex pathways between cervical and lumbar cord both in animals (Miller et al. 1973; Danner et al. 2018) and in humans (Meinck and Piesiur-Strehlow 1981; Zehr et al. 2001). However, it remains unclear if such inter-limb facilitation of the spinal reflex excitability occurs when continuous electrical stimulation-evoked contraction of muscles is applied instead of voluntary contraction.

Neuromuscular electrical stimulation (NMES) can be used to generate muscle contraction by depolarizing axons of alpha motor neurons beneath the electrodes placed on the skin surface over the muscles or nerves, which provides a rich afferent input to central nervous system without voluntary effort (Burke et al. 1983; Bergquist et al. 2011). Most afferent information is sent to the central nerve system simultaneously, stimulating Ia afferents, Ib afferents, and cutaneous nerves (Burke et al. 1983; Bergquist et al. 2011). A previous study showed that on–off NMES changed the spinal reflex excitability of stimulated lower limb muscles, inducing the increase of presynaptic inhibition onto Ia afferent terminals (Grosprêtre et al. 2018). Similarly, H-reflex excitability was facilitated after NMES (Kitago et al. 2004). These studies imply that NMES could change the spinal reflex excitability. However, no study has systematically evaluated the inter-limb effect during continuous electrical stimulation of muscles and nerves. Previous studies utilizing Jendrássik maneuver typically performed hand grip tasks, while evaluating lower limb reflexes. Similarly, NMES was applied over the medial nerve in this study to activate wrist flexor muscles, which are activated during hand griping. Moreover, Ia afferents are more activated by NMES over a nerve trunk compared to stimulation over the muscle belly (Bergquist et al. 2011). If short-term NMES on the median nerve affects the spinal reflex excitability of lower limb muscles, it would imply that peripheral afferents play a role in the inter-limb effect during Jendrássik maneuver facilitation. Therefore, in this study, we used NMES to investigate how electrical stimulation on the median nerve affects the inter-limb spinal reflex excitability of lower limb muscles. Furthermore, it has previously been demonstrated that NMES combined with voluntary contraction has greater effect on the central nervous system (Thompson et al. 2006; Lagerquist et al. 2012). We also used NMES with voluntary contractions to investigate possible inter-limb facilitation effects.

Previous studies have typically investigated a single muscle (i.e., soleus) to investigate remote effect facilitation of lower limb muscles during bilateral upper limb contraction tasks (Dowman and Wolpaw 1988; Zehr and Stein 1999; Gregory et al. 2001). Therefore, it is also still unclear whether neural signals generated by unilateral voluntary contractions or unilateral NMES application to arm muscles spreads bilaterally or unilaterally over inter-limb segments (i.e., whether there is a remote crossed effect). Transcutaneous spinal cord stimulation (tSCS) can be used to evoke spinal reflexes in multiple lower limb muscles (Courtine et al. 2007; Minassian et al. 2007; Masugi et al. 2019). A single electrical stimulus over the lumbosacral enlargement generates reflex responses, activating the dorsal roots of the spinal nerves. Similar to H-reflex, the responses evoked by tSCS are thought to reflect the excitability of monosynaptic responses (Courtine et al. 2007; Minassian et al. 2007). Therefore, in this study, we used tSCS to investigate the spinal reflex excitability of bilateral multiple lower limb muscles simultaneously during unilateral upper limb contractions.

The objective of current study was to investigate how upper limb contractions generated by NMES of the median nerve and voluntary muscle contractions affect the spinal reflex excitability of lower limb muscles bilaterally: (1) sensory-level NMES; (2) motor-level NMES; and (3) voluntary contraction. Moreover, the second objective of this study was to investigate how combining NMES and voluntary contraction of the upper limb may affect facilitation of spinal reflex excitability: (4) voluntary contraction and sensory-level NMES; (5) voluntary contraction and motor-level NMES. Afferent feedback during voluntary contractions plays an important role in modulating the spinal reflex excitability (Brooke et al. 1997). Moreover, it has been shown that stimulation of the median nerve facilitated the soleus H-reflex (Kagamihara et al. 2003). Therefore, we hypothesized that continuous NMES at intensities that evoke muscle contractions (i.e., motor-level NMES) without voluntary drive can send strong afferent inputs to the central nervous system (i.e., spinal intraneuronal networks) and alter the spinal reflex excitability of lower limb muscles bilaterally, as with the inter-limb reflex facilitation during volitional contraction. Moreover, we also hypothesized that providing additional volition drive during NMES will produce larger effect than voluntary contraction only.

## Methods

### Participants

Eleven able-bodied, right-handed males participated in the study (age: 25.6 ± 3.6 years, height: 173.6 ± 5.2 cm, weight: 69.8 ± 9.8 kg). All participants had no history of neuromuscular and sensory disorders. All participants gave written informed consent in accordance with the Declaration of Helsinki. The experimental procedures were approved by the local institutional ethics committee at The University of Tokyo.

### Protocol

**Fig. 1:**
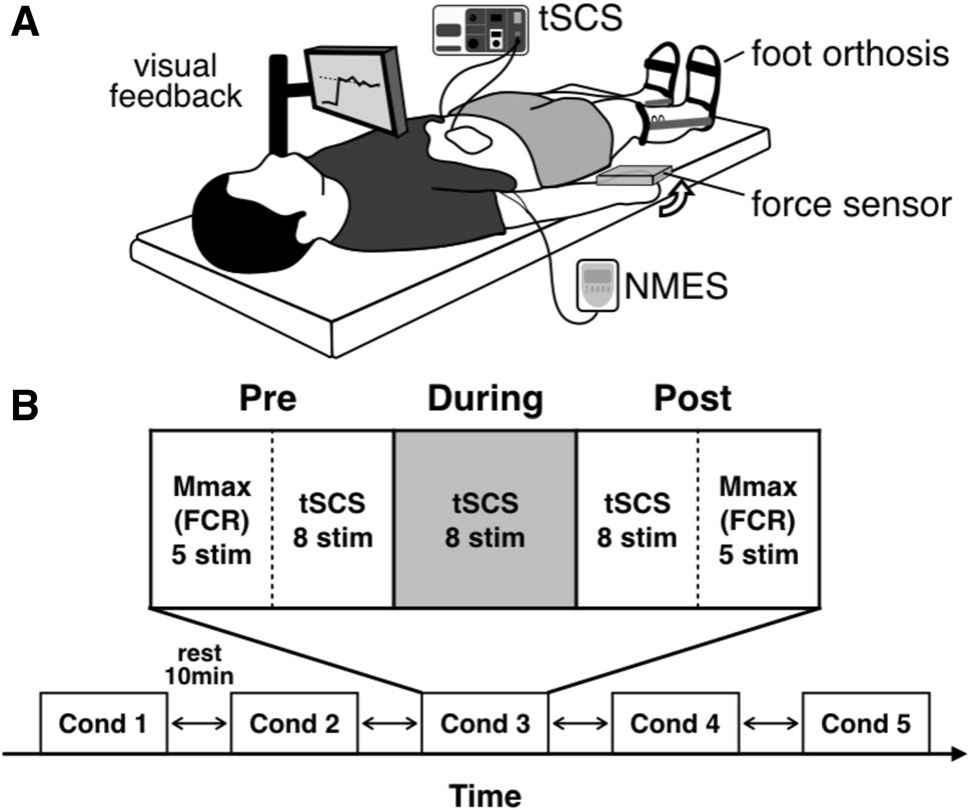
Experimental setup and protocol of this study: **a** participants were in the supine position on the bed. Both legs were fixed by an orthosis for the duration of the experiments to avoid movements. Real-time visual feedback for the wrist flexion force was provided on the monitor. **b** Maximum motor response (*M*_max_) of flexor carpi radialis (FCR) and transcutaneous spinal cord stimulation (tSCS) responses were assessed before (Pre) and after (Post) each condition. tSCS responses were also measured during (During) each condition. Five conditions were performed in a random order and 10 min rest was set between conditions

During the experiment, participants remained in the supine position to elicit stable spinal reflexes (Danner et al. 2016) (Fig. 1a). First, maximal voluntary contraction (MVC) force of right wrist flexors was measured by asking the participants to flex their right wrist as hard as possible and to relax other upper limb muscles, while isometric force was measured using a strain gauge sensor (LCB03K025L, A&D Company Limited, Japan). Two MVC trials were performed, with at least 10 s between trials, and averaged to determine the MVC force level for each subject. Prior to performing the MVC trials, all subjects were first warmed up and practiced the experimental tasks. Moreover, if one of the two trials resulted in a much higher force, an additional trial was performed and the average of the two highest trials was selected as the MVC level. Participants then performed the five experimental conditions in a random order: (1) sensory stimulation (SS)—NMES was applied to the right median nerve at the intensity which was set to 1 mA below the stimulation that produced palpable contractions, such that it did not evoke muscle contractions and/or wrist flexion force (see NMES section); (2) motor stimulation (MS)—NMES was applied to the right median nerve at the intensity which elicited 10% of MVC wrist flexion (see NMES section); (3) voluntary contraction (Vol)—participants were asked to flex the right wrist at the 10% of MVC force voluntarily; (4) sensory stimulation and voluntary contraction (SS + Vol)—during sensory stimulation, which did not produce muscle contractions and/or wrist flexion, participants were asked to contract the right wrist at 10% of MVC force voluntarily; (5) motor stimulation and voluntary contraction (MS + Vol)—in addition to NMES applied at the motor level, participants were asked to actively maintain the force level by voluntary flexing their wrist if necessary to ensure that the visual feedback target was always maintained at 10% of MVC level during the trial (NOTE: during the MS condition, participants did not have force visual feedback). The force produced by the right wrist flexors and/or NMES was displayed on a computer monitor so that participants was able to maintain 10% of MVC wrist flexion force using visual feedback. Voluntary contractions were matched to NMES condition by setting the intensity of stimulation to evoke muscle contractions at 10% of MVC force level (see Neuromuscular electrical stimulation section). Visual feedback was provided to participants during voluntary contraction conditions only (i.e., Vol, SS + Vol, and MS + Vol). Each intervention time was applied for 70 s and a resting period of at least 10 min was set between the conditions. Each intervention was applied unilaterally such that we could investigate whether inter-limb facilitation of spinal reflexes would have crossed effects bilaterally. Spinal reflexes of lower limb muscles and maximum motor response (*M*_max_) of right flexor carpi radialis (FCR) (see Spinal reflexes and *M*_max_ sections) were evoked: (i) pre; (ii) during; and (iii) post each intervention (Fig. 1b).

### Neuromuscular electrical stimulation (NMES)

NMES was delivered to the median nerve in the right upper limb using a portable, constant-current electrical stimulator (Rehab, Chattanooga, DJO Global, USA). Biphasic rectangular stimulation waves were applied with a 400 µs pulse width and 20 Hz frequency via circular surface electrodes (2 cm diameter) positioned on the brachium (cathode) and on the motor point (anode), which was searched by the experimenter using a pen electrode to locate a motor point that evoked wrist flexion effectively. Pulse width and frequency used in this study were determined based on previous studies, which suggested that these parameters can effectively stimulate the sensory nerves (i.e., produce afferent input), but remain fatigue resistant (Kesar and Binder-Macleod 2006; Bergquist et al. 2011). Prior to the start of the experiment, the experimenter identified the motor threshold of NMES by gradually increasing the stimulation amplitude with 1 mA increments and checking for palpable wrist flexion contractions. The sensory stimulation (SS) amplitude was set the intensity that was 1 mA below the motor threshold, such as not to produce any wrist flexion (i.e., muscle contractions) but to remain above the perceptual threshold (i.e., participants were asked if they could feel the stimulation). The motor stimulation (MS) amplitude was set such as to produce 10% of MVC of the wrist flexion force. It was ensured that the stimulation amplitude for the SS and MS conditions was at a tolerable level for all participants, and they were kept constant throughout the experiment.

### Spinal reflexes

Spinal reflexes were elicited in multiple lower limb muscles simultaneously using tSCS with a constant-current electrical stimulator to apply a single monophasic pulse on the lumbar area of the spine, with the pulse width set to 1 ms (Digitimer, DS7A, UK). Responses recorded from tibialis anterior (TA), soleus (Sol), vastus medialis (VM), and biceps femoris (BF) muscles bilaterally (ipsilateral: iTA, iSol, iVM, and iBF; contralateral: cTA, cSol, cVM, and cBF) using surface electromyography (EMG) electrodes (Ag/AgCl; Vitrode F-150S, Nihon Koden, Japan). A long reference electrode was placed around the circumference of the knee (Ag/AgCl; 45400-SK, GE Healthcare, US). The anode electrode (5 × 5 cm) was positioned on the midline of the abdomen and the cathode electrode (7.5 × 10 cm) was placed on the lumbar spine. The optimal site of the cathode electrode was determined based on the location that induced larger responses simultaneously in all recorded lower limb muscles (i.e., T12/L1: *n* = 1; L1/L2: *n* = 10; L2/L3: *n* = 1) (Masugi et al. 2017). After the optimal site was decided, the stimulation intensity was set based on obtaining the recruitment curve of responses for each participant (Milosevic et al. 2018; Masugi et al. 2019). Participants were prompted to keep their heads stable throughout the experiment to not change the stimulus location (Courtine et al. 2007). Moreover, participants wore a foot orthosis to prevent leg movements during the experiment (Fig. 1a). The intensity for eliciting the spinal reflexes was adjusted to evoke responses on the ascending part of the recruitment curve in all recorded muscles simultaneously and it was kept constant throughout the experiment and in all experimental conditions (Masugi et al. 2017; Milosevic et al. 2018). Prior to starting the experiment, based on previous studies (Masugi et al. 2017; Milosevic et al. 2018), a paired-pulse stimulus was applied by delivering two stimulation pulses, with a 50 ms inter-pulse interval, to test homosynaptic depression of the evoked responses. Confirming suppression of the second evoked responses would indicate that spinal reflexes were evoked from afferent fibers by stimulating the dorsal root of the spinal column, rather than evoking motor responses directly (Courtine et al. 2007). A total of eight paired-pulse stimuli were elicited for each participant, with 10 s intervals between each pair, and their responses were averaged. Moreover, during the experiment, a total of eight single-pulse stimuli were elicited for each experimental condition and at each time interval (i.e., (i) pre; (ii) during; and (iii) post the intervention) with 10 s intervals between the stimuli.

### Maximum motor response (*M*_max_)

Maximum motor response (*M*_max_) of the right (stimulated) FCR muscle was representatively recorded before and after each experimental condition by stimulating the median nerve using surface electrodes placed between the biceps brachii and brachialis. The anode was placed proximally and the cathode distally, with 1.5 cm separation. Five *M*_max_ responses were elicited before and after each experimental condition, with 5 s interval between the stimuli (Crone et al. 1999). The *M*_max_ responses can be used to check for the effects of fatigue of different experimental conditions and chronologically for the duration of the experiment (Sacco et al. 1997; Crone et al. 1999; Obata et al. 2015).

### Data analysis

The tSCS- and *M*_max_-evoked responses were pre-amplified (× 1000) and band-pass filtered at 15–3000 Hz (Masugi et al. 2016; Milosevic et al. 2018) using a biosignal amplifier (MEG-6108, Nihon Kohden, Japan). All data were sampled at 4000 Hz with an analog-to-digital converter (Power lab/16SP, AD Instruments, Australia) and saved on the computer for off-line analysis. The tSCS and *M*_max_ peak-to-peak amplitudes were calculated offline using a custom-written code (Matlab, Mathworks Inc., USA) without any additional processing (Masugi et al. 2017; Milosevic et al. 2018). To calculate the peak-to-peak amplitudes, the latency of tSCS response was first defined based on a previous study (Courtine et al. 2007). Specifically, in proximal lower limb muscles (i.e., VM and BF), tSCS amplitude was defined as the peak-to-peak amplitude which appeared 15 ms after the tSCS stimulus and, in distal lower limb muscles (i.e., TA and Sol), tSCS amplitude was defined as the peak-to-peak amplitude which appeared 20 ms after tSCS stimulus. All latencies and results were confirmed visually. Prior to statistical analysis, spinal reflex responses (i.e., tSCS) were normalized by the amplitude obtained before each experimental condition (i.e., pre). Moreover, *M*_max_ responses were normalized by the amplitude obtained before the first condition, which was performed for each participant.

### Statistics

Normality of data was first tested using the Shapiro–Wilk test and the tests indicated that not all data were normality distributed. Thus, Friedman test, a non-parametric equivalent for repeated-measure analysis of variance (ANOVA), was used to test differences of spinal reflex amplitudes before, during, and after each intervention in each muscle to find the change of *M*_max_ throughout the experiment (i.e., temporal change in *M*_max_ for the duration of the experiment) and to test differences of background EMG (calculated in the 50 ms window before the tSCS stimulus) between pre, during, and post each intervention in each muscle. When Friedman test resulted in a significant effect, Wilcoxon signed-rank tests with Bonferroni correction were used to compare the mean values. Moreover, Wilcoxon signed-rank test was also used to compare the first and second responses of the paired-pulse stimulation and the *M*_max_ amplitudes before and after each intervention. Significant level was set at *p* < 0.05 for all tests.

## Results

### Maximum motor responses (*M*_max_)

**Fig. 2:**
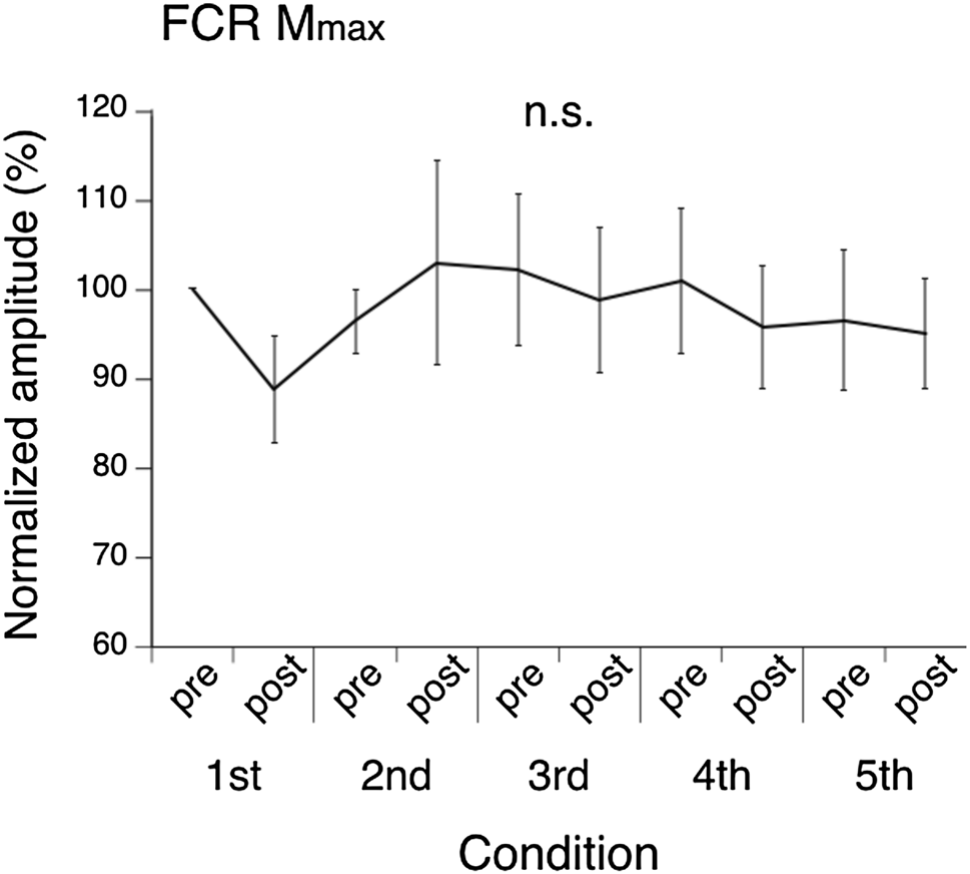
Results of the maximum motor response (*M*_max_) peak-to-peak amplitude mean and standard error (SE) for the flexor carpi radialis (FCR). Responses were measured before and after each condition. The amplitudes of responses were arranged in chronological order and were normalized by the amplitude obtained before the first condition (first Pre). Legend: n.s. *p* > 0.05

Results of the maximum motor response (*M*_max_) of the FCR muscle throughout the experiment are shown in Fig. 2. Statistical comparisons showed that there is no main effect of *M*_max_ during the whole experiment (i.e., temporal changes during the experiment). Moreover, *M*_max_ amplitude before and after each intervention was also not significantly different. Overall, the result suggested that wrist flexor muscles were not affected by peripheral fatigue through the experiment (Crone et al. 1999).

### Paired-pulse stimulation

**Fig. 3:**
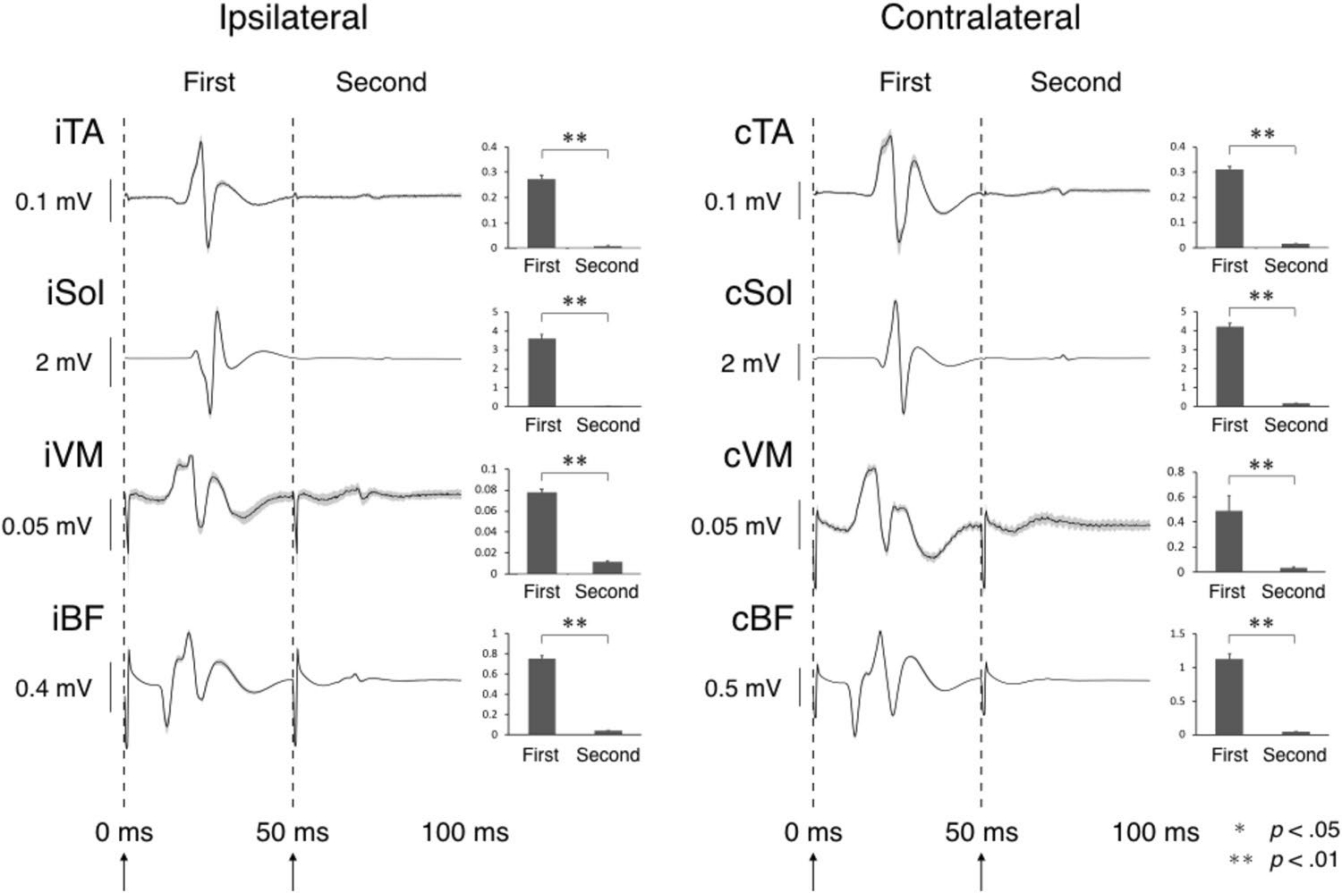
Responses elicited by the paired-pulse transcutaneous spinal cord stimulation (tSCS). The time series plots represent the mean ± SD of eight repeated responses for one representative subject. Bar graphs represent peak-to-peak amplitude mean and standard error (SE) of all participants. The first and second stimulus was applied 50 ms apart. Evoked responses were recorded bilaterally in the tibialis anterior (iTA and cTA), soleus (iSol and cSol), vastus medialis (iVM and cVM), and biceps femoris (iBF and cBF) muscles. *p < 0.05; **p < 0.01

Representative evoked responses of the tSCS paired-pulse stimulus protocol are shown in Fig. 3. Statistical comparisons of the average responses across all participants indicated that the first stimulus evoked significantly larger responses compared to the second stimulus in all recorded muscles (Fig. 3). These results suggest that tSCS elicited spinal reflexes of lower limb muscles bilaterally (Courtine et al. 2007; Minassian et al. 2007).

### Spinal reflexes

**Fig. 4:**
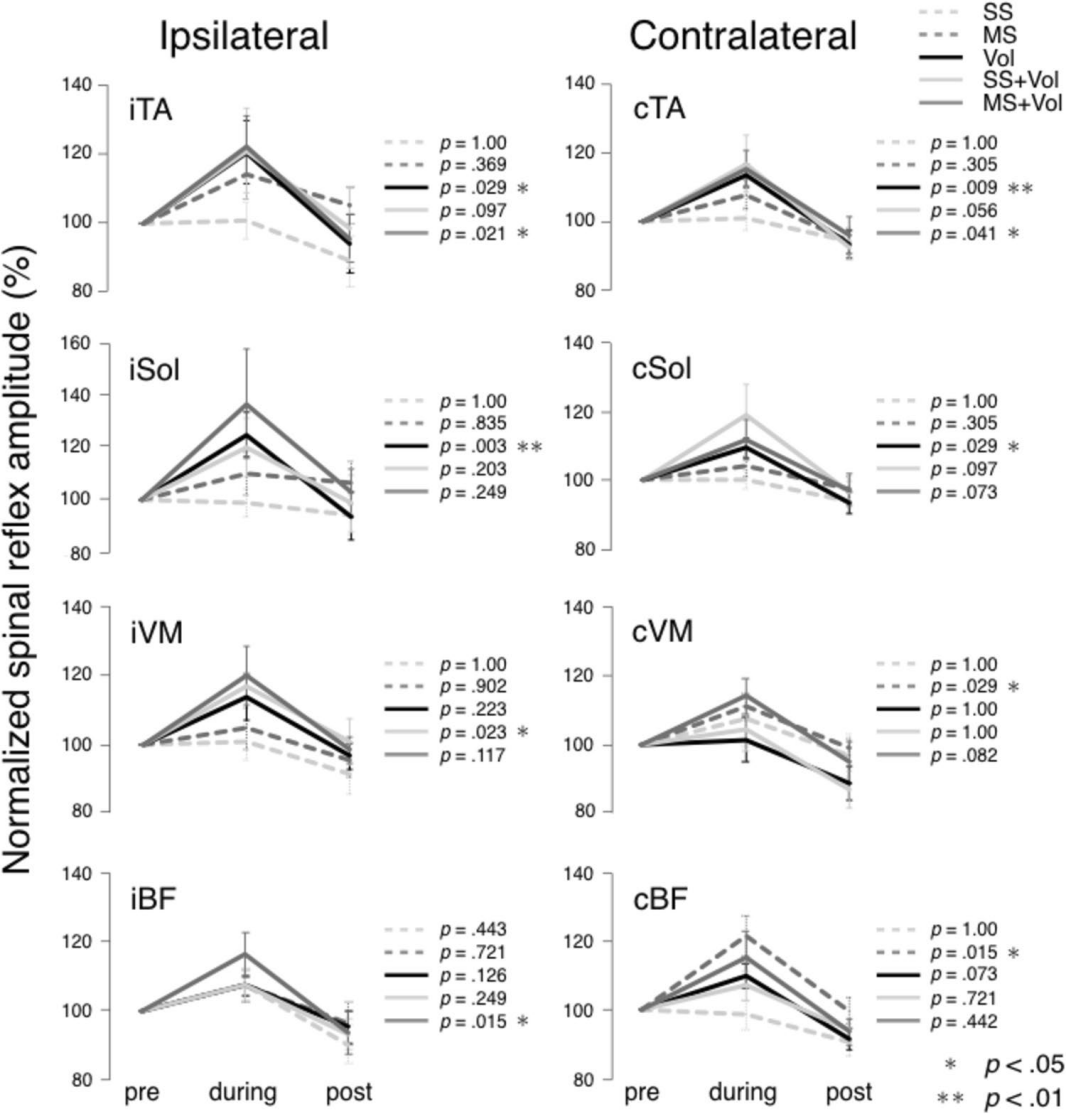
Results of the spinal reflex peak-to-peak amplitude mean and standard error (SE) elicited using transcutaneous spinal cord stimulation (tSCS) for the bilateral tibialis anterior (iTA and cTA), soleus (iSol and cSol), vastus medialis (iVM and cVM), and bicep femoris (iBF and cBF). Responses were measured before, during, and after the intervention (pre, during, and post, respectively). The amplitude of the spinal reflexes was expressed as a percentage of baseline assessment (Pre) for each muscle in the five conditions: (1) sensory-level stimulation (SS) (light gray dotted line); (2) motor-level stimulation (MS) (gray dotted line); (3) voluntary contraction (Vol) (black line); (4) sensory-level stimulation and voluntary contraction (SS + Vol) (light gray line); (5) motor-level stimulation and voluntary contraction (MS + Vol) (gray line). Statistical comparisons examined differences between the baseline amplitude and the mean amplitude during and after the interventions. *p < 0.05; **p < 0.01

For all muscles, background EMG activity was not significantly different between pre, during, and post for each intervention (all *p* > 0.05). Results comparing spinal reflexes during different experimental interventions are summarized in Fig. 4. Sensory-level NMES (SS) intervention had no significant effect in any muscle. Voluntary contraction (Vol) facilitated TA and Sol responses bilaterally during the intervention. Moreover, voluntary contractions with motor-level stimulation (MS + Vol) facilitated TA responses bilaterally, as well as the spinal reflex of iBF during the intervention. Sensory-level NMES and voluntary contraction (SS + Vol) facilitated iVM responses during the intervention. Motor-level NMES (MS) facilitated contralateral thigh muscle (cVM and cBF) spinal reflexes during the intervention. However, in all muscles and interventions, the amplitudes of the spinal reflexes after the intervention were not significantly different compared to before the interventions.

## Discussion

In the current study, we investigated whether unilateral short-term application of NMES on the median nerve, unilateral voluntary muscle contractions of the upper limb, or their combined action, affects the spinal reflex excitability of the lower limb muscles bilaterally. The tSCS stimuli were used to evoke posterior-root responses (Courtine et al. 2007; Minassian et al. 2007; Masugi et al. 2019). Since the second response was significantly suppressed compared to the first response in the paired-pulse stimuli protocol (Fig. 3), our results confirmed the homosynaptic (post-activation) depression of the second response (Courtine et al. 2007; Minassian et al. 2007; Masugi et al. 2017). The refractory period of posterior-root muscle reflex responses, therefore, demonstrated that the evoked responses represent spinal reflex excitability (Minassian et al. 2004, 2007). Specifically, our results showed that unilateral weak muscle contraction of wrist flexors (i.e., Vol) facilitated the spinal reflex excitability of the lower limbs bilaterally (i.e., iTA, iSol, cTA, and cSol), similar to previous studies (Miyahara et al. 1996; Masugi et al. 2019). In addition, unilateral voluntary contraction combined with motor-level electrical stimulation (i.e., MS + Vol) facilitated the spinal reflex excitability of the lower limbs bilaterally (i.e., iTA, cTA, and iBF). In all protocols, after the experimental condition (i.e., post), spinal reflex excitability immediately returned to baseline levels. We also found that muscle contractions generated without voluntary commands by applying motor-level NMES alone (i.e., MS) affected contralateral thigh muscles (i.e., cVM and cBF), but not the distal ankle muscles. On the other hand, cutaneous afferents evoked by sensory-level NMES (i.e., SS) had no effect on the interlimb spinal reflex excitability. Moreover, it has previously been demonstrated that *M*_max_ amplitude can decrease as a result of a muscle fatigue (Sacco et al. 1997; Milosevic et al. 2018). However, the effects in our study were not caused by peripheral fatigue, since *M*_max_ in the FCR muscle was not significantly affected during the experiments and by each experimental intervention. Therefore, our results can be attributed to facilitation of inter-limb spinal reflex circuits. A specific discussion of different experimental conditions follows.

### Remote effect during voluntary muscle contractions

It is well known that strong contractions of upper limb muscles during the Jendrássik maneuver can facilitate the H-reflex excitability of the soleus muscle (Landau and Clare 1964; Dowman and Wolpaw 1988). Moreover, the amount of facilitation can increase proportionally to the strength of the handgrip during upper limb contractions (Bussel et al. 1978; Miyahara et al. 1996). Specifically, Bussel et al. (1978) reported that when handgrip was increased to 25%, 50%, and 100% of the maximal effort force, the amplitude of H-reflexes was also increased proportionally. Our current results confirmed, and extended, these previous findings by demonstrating that relatively small (i.e., 10% of maximal effort) voluntary contraction of upper limbs (Vol) can also facilitate the spinal reflex excitability of the lower limb muscles (Fig. 4). This result suggests that even single joint movements caused by weak muscle contractions are sufficient to potentiate the spinal reflex networks in a remote limb.

The mechanism of interlimb facilitation during voluntary flexion of the wrist, is likely to be explained by the central motor command facilitation. Specifically, it is well known that motor evoked potential (MEP), elicited by transcranial magnetic stimulation (TMS) over the motor cortex to evaluate the corticospinal excitability, are facilitated by remote muscle activations (Kawakita et al. 1991; Péréon et al. 1995; Sasaki et al. 2018). MEP typically does not change during fatiguing contraction of the remote muscles, although the output force declines (Tazoe et al. 2009). Tazoe et al. (2009) have also investigated cervicomedullary motor-evoked potential (CMEP), which evaluates the direct effect of corticospinal tract in the subcortical mechanisms, on the hand muscles during ipsilateral knee extension. While MEP responses were facilitated, remote effect of CMEP responses was not observed during the contractions of the remote muscle (Tazoe et al. 2009). These results suggest the possibility that the effect by contracting remote limb muscles may derive from the central motor commands (i.e., cortical activations). In our study, the voluntary flexion of the wrist facilitated the spinal reflex excitability of the distal ankle muscles (Fig. 4). It has been suggested that there is a connectivity at the cortical level between the wrist and the distal ankle muscles (Ehrsson et al. 2000). Specifically, using positron emission tomography (PET), it has been demonstrated that the supplementary area related to wrist movement is partly overlapped with the area related to ankle movements (Ehrsson et al. 2000). Therefore, planning to move the wrist may be able to activate cortical neurons not only for wrist but also for the ankle muscles. Taken together, it is possible that the central motor commands activating the wrist facilitate the spinal reflex excitability of distal ankle muscles by simultaneously activating the cortical areas controlling the ankle joint.

Furthermore, the remote effect by voluntary muscle contraction of the wrist is transmitted to distal ankle muscles, rather than the more proximal thigh muscles. Previous studies have shown that simultaneous movement of wrist and ankle in the sagittal plane are strongly constrained by the movement direction (Baldissera et al. 1982; Hiraga et al. 2004; Nakagawa et al. 2015). Specifically, in the sagittal plane, it is easier to move ipsilateral wrist and ankle in the same direction simultaneously than in the opposite direction. In short, the ankle movement is constrained with the ipsilateral wrist movement. Such inter-limb coordination shows functional connectivity of wrist and ankle muscles. Our current result demonstrating neural connectivity may derive from this functional connectivity between the wrist and the ankle joints. Group Ia afferents in the lower limb muscles have both homonymous connections and heteronymous connections, which implies that different muscle groups share common intraneuronal pathways that affect motoneurons of other muscles in the same limb across different joints (Harrison et al. 1983; Meunier and Pierrot-Deseilligny 1998). However, our current study also provides evidence that descending signals generated by voluntary wrist flexion (Vol) were not only sent to common interneurons, but that they were transmitted to individual motoneurons of lower limb muscles. Finally, our results also showed that unilateral voluntary activation of wrist flexors facilitated both ipsilateral and contralateral spinal reflexes of the lower limbs. Interhemispheric inhibition is well known as a mechanism that can suppress brain activations, during which the activation of unilateral hemisphere inhibits the activation of the contralateral brain region (Ohtsuki 1983; Oda and Moritani 1994; Daffertshofer et al. 2005). Therefore, activation between hemispheres has inhibitory effects reciprocally. In contrast, in our study, the spinal reflex excitability was facilitated bilaterally. Hence, bilateral facilitation by unilateral remote limb muscle activation seems to be related to the spinal intraneuronal mechanism. Although the specific mechanisms still remain unclear, our study may suggest that unilateral descending commands to the forearm are sent to bilateral interneurons of distal ankle muscles.

Previous studies reported that the modulation of presynaptic inhibition at Ia terminals could contribute to the facilitation on H-reflex during Jendrássik maneuver (Dowman and Wolpaw 1988; Zehr and Stein 1999; Gregory et al. 2001). It has been suggested that H-reflexes are not strictly monosynaptic and that oligosynaptic contributions may be involved (Burke et al. 1983; Gregory et al. 2001). Descending commands following voluntary contractions may modulate the presumed interneurons mediating polysynaptic circuitry of a remote limb muscle (Tazoe et al. 2005).

### Effects of NMES on the inter-limb facilitation

Motor-level NMES (i.e., MS) facilitated the spinal reflex excitability of lower limbs, as we hypothesized, but the facilitation effect appeared only in contralateral thigh muscles (i.e., cVM and cBF) (Fig. 4). However, sensory stimulation of the cutaneous circuits elicited by sensory-level NMES (i.e., SS), which produce no muscle contractions, did not have the inter-limb facilitation effects. This result implies that cutaneous information evoked by sensory stimulation is not sufficient to change the spinal reflex excitability of inter-limb muscles (Milosevic et al. 2018). Hundza et al. (2012) reported that the inhibitory effect on the Sol H-reflex during active arm cycling was not observed during passive arm cycling. Therefore, it is considered that abundant Ia afferents are needed to produce the inter-limb facilitation effect. Nevertheless, this effect was observed only in contralateral proximal lower limb muscles. In walking, the diagonal upper limb and lower limbs are moved synchronously. To maintain the posture during walking, thigh muscles can be more sensitive to afferent information from the contralateral upper limbs. This may be why spinal reflex excitability of contralateral proximal lower limb muscles was facilitated during motor-level NMES. Moreover, the stimulus intensity which used in our current study was set at 10% of MVC wrist flexion force. Afferent information evoked by motor-level NMES might not be sufficient to affect other muscles (i.e., ipsilateral lower limb muscles and contralateral distal lower limb muscles). However, only a few studies have investigated inter-limb effects of NMES in the literature. Further research is needed to clarify the characteristics of the inter-limb effects evoked by NMES.

Change of spinal reflex excitability of ankle dorsiflexors (i.e., TA muscles) was observed bilaterally even during voluntary muscle contraction assisted with motor-level NMES (i.e., MS + Vol), but in the plantar flexors (i.e., Sol muscle), spinal reflex excitability was not modulated (Fig. 4). These effects are different from those induced during voluntary contraction of muscles. When muscles are contracted with the assistance of motor-level NMES during voluntary contractions, voluntary effort is arguably smaller compared to when muscles are just voluntarily contracted. Our results may, therefore, reflect the difference in the degree of involvement of the central motor drive. Specifically, the TA muscle is known to have a stronger connectivity to the corticospinal pathway compared to the Sol muscle (Morita et al. 2000; Lagerquist et al. 2012). Thus, it was easier to affect the TA muscle compared to the Sol muscle in the corticospinal circuits. This may, therefore, suggest that the contribution of central motor commands to the inter-limb facilitation had an effect. Meanwhile, although voluntary muscle contraction assisted with sensory-level NMES (i.e., SS + Vol) needed more voluntary effort compared to MS + Vol condition, it facilitated the spinal reflex excitability of only ipsilateral VM muscle. In addition, the spinal reflex excitability of ipsilateral BF was facilitated during motor-level NMES with voluntary contractions (Fig. 4). The amount of afferent information during NMES with voluntary contractions (i.e., SS + Vol and MS + Vol) is larger compared to voluntary contractions alone. Therefore, when NMES is utilized in combination with the voluntary drive, the central nervous system receives more afferent feedback than expected from the voluntary drive. Moreover, the sensory nerve is discharged relatively synchronously during NMES (Bergquist et al. 2011). Hence, afferent information evoked during NMES with voluntary contraction is sent to the central nervous system with a rhythm different from voluntary contraction alone. These mismatches in the amount and the rhythm of afferent information could make a unique effect on the spinal reflex excitability, which is contrary to our hypothesis. This implies that the inter-limb effect derived from the voluntary drive could be altered by unusual afferent information. Therefore, further work is warranted to understand mechanisms of NMES with voluntary contractions.

### Implications for rehabilitation

In rehabilitation after spinal cord injury, significantly larger improvements of walking function and the cervicolumbar connectivity were found in the training group that included combined arm and leg cycling compared to the group that trained using only leg cycling (Zhou et al. 2018a, b). These studies recommended active use of arm involvement during training to maximize improvements of walking function, which is directly aligned with our current study findings. Specifically, our present study supplements this suggestion by showing that active/voluntary effort is important for facilitating the inter-limb connectivity, a mechanism proposed in clinical observations. Therefore, training of walking function should attempt to simultaneously involve upper limbs voluntarily when possible and/or with NMES activation of muscles.

### Limitations

Some limitations should be considered in the present study. First, Courtine and colleagues reported that spinal reflex responses of the TA muscle evoked by tSCS could be contaminated by Sol muscle activity (Courtine et al. 2007). Though the spinal reflex excitabilities of both TA muscle and Sol muscle were facilitated during voluntary wrist flexion, the observed TA muscle facilitation could also be affected by crosstalk to the Sol muscle. However, since background EMG of all lower limb muscles was relatively low, the crosstalk effect might not be very significant. Second, the spinal reflex responses evoked by tSCS were not controlled (normalized) by *M*_max_ amplitude. It is unfeasible to consistently evoked M-wave using tSCS in multiple muscles simultaneously due to a large stimulus current requirement. However, the stimulation intensity of tSCS was adjusted to be on the ascending part of the recruitment curve in all recorded muscles simultaneously. Moreover, baseline amplitude was measured before each intervention (Fig. 1b) and it was used to normalize the spinal reflex responses for each intervention as in previous reports (e.g., Milosevic et al. 2018; Masugi et al. 2019) (see Data analysis). Third, pulse-width and stimulation frequency (i.e., 400 µs, 20 Hz, respectively) were set to elicit sensory volley efficiently, while minimizing effects of rapid fatigue (Bergquist et al. 2011). However, it has been also reported that wider pulse width and higher frequency of NMES (1 ms, > 80 Hz, respectively) can more effectively depolarize large sensory diameter afferents (Collins 2007; Neyroud et al. 2019). Therefore, future studies should carefully select the stimulation parameters to activate the sensory pathway while investigating inter-limb facilitation effects.

## Conclusion

We used NMES to the median nerve unilaterally to investigate the effect of peripheral afferent information on the spinal reflex excitability of lower limbs, compared to voluntary unilateral muscle contractions of the upper limb. Our results showed that voluntary flexion of the wrist facilitated the spinal reflex excitability of distal ankle muscle bilaterally. Meanwhile, the spinal reflex excitability of contralateral thigh muscles was facilitated by wrist flexion induced by motor-level NMES. Overall, the present results suggest that the central motor commands are important for the facilitation of spinal reflex excitability of remote limb muscles by voluntary muscle contractions and imply that distal ankle muscles have a stronger connectivity with the forearm compared to the thigh muscles in the interlimb facilitation. The afferent information evoked by NMES on upper limb has different effects compared to voluntary muscle contractions on the spinal reflex excitability of lower limbs. Nonetheless, involvement of upper limb muscles either by voluntary contractions or NMES can likely help in rehabilitation of walking.
